# Serial Changes in CSF Volume Proportion on Brain CT and 6-Month Neurologic Outcomes After Cardiac Arrest: A 72–96-h Landmark Cohort Study

**DOI:** 10.3390/jcm15186978

**Published:** 2026-09-09

**Authors:** Seungho Lee, Jung Soo Park, Hyonshik Ryu, Jin Hong Min, Changshin Kang, Yeonho You, Byung Kook Lee

**Affiliations:** 1 Department of Emergency Medicine, College of Medicine, Chungnam National University, Daejeon 35015, Republic of Korea; leesh4169@cnuh.co.kr (S.L.); laphir@cnu.ac.kr (J.H.M.); rosc@cnu.ac.kr (C.K.); yyo1003@naver.com (Y.Y.); 2 Department of Emergency Medicine, Chungnam National University Hospital, Daejeon 35015, Republic of Korea; 3 Department of Emergency Medicine, Chonnam National University Hospital, Chonnam National University Medical School, Gwangju 58128, Republic of Korea; bbukkuk@hanmail.net

**Keywords:** cardiac arrest, prognosis, brain edema, cerebrospinal fluid, hypoxic–ischemic brain injury, computed tomography

## Abstract

**Background**: Single-time-point cerebrospinal fluid (CSF) volume proportion (pCSFV) has shown limited prognostic utility after out-of-hospital cardiac arrest (OHCA). We hypothesized that serial within-patient change in pCSFV (ΔpCSFV), rather than absolute values, would reflect progression of neurologic injury. **Methods**: This retrospective 72–96-h landmark cohort study included comatose adult OHCA survivors who underwent brain computed tomography (CT) within 6 h (early) and at 72–96 h (delayed) after return of spontaneous circulation. pCSFV was quantified using Hounsfield-unit-based volumetry. ΔpCSFV was calculated as delayed pCSFV minus early pCSFV. The primary outcome was poor 6-month neurologic outcome (Cerebral Performance Category 3–5). **Results**: Among 125 patients, 63 (50.4%) had poor neurologic outcomes. Early and delayed pCSFV did not differ significantly between outcome groups, whereas ΔpCSFV was lower in the poor-outcome group (*p* < 0.001). In the five-parameter multiple-imputation model, ΔpCSFV was associated with poor outcome (adjusted odds ratio [aOR] 0.684 per 1 percentage-point increase, 95% confidence interval [CI] 0.494–0.947; *p* = 0.022), with a consistent result in the complete-case sensitivity analysis (aOR 0.648, 95% CI 0.446–0.940; *p* = 0.022). However, this association was attenuated after additional adjustment for early pCSFV (aOR 0.728, 95% CI 0.462–1.146; *p* = 0.170). Adding ΔpCSFV to the baseline model did not significantly improve discrimination (area under the curve 0.962 vs. 0.955; *p* = 0.292). **Conclusions**: ΔpCSFV may provide complementary information on serial CSF-space changes but should not be interpreted as a baseline-independent marker. These findings apply only to patients who survived and underwent delayed CT at 72–96 h. The observed data-derived cutoff is exploratory and requires external validation before any clinical application.

## 1. Introduction

Hypoxic–ischemic brain injury (HIBI) remains the leading cause of death and long-term disability among comatose survivors of out-of-hospital cardiac arrest (OHCA) who achieve return of spontaneous circulation (ROSC) [1,2,3]. Post-arrest brain injury is not static: Cerebral edema and related imaging abnormalities evolve from the immediate post-resuscitation phase into the subacute period, with ongoing swelling that may continue for hours to days after ROSC [3,4,5]. This temporal evolution has direct implications for neuroimaging-based prognostication because scans obtained very early after ROSC may underestimate the eventual injury burden, whereas delayed or serial assessment may better reflect cumulative edema progression.

According to the Monro–Kellie doctrine, intracranial volume is constrained by the cranial vault, such that increases in brain tissue volume must be compensated by reductions in intracranial blood and cerebrospinal fluid (CSF) [6]. In post-arrest HIBI, progressive brain swelling should therefore manifest as displacement or loss of visible CSF space on computed tomography (CT). Consistent with this framework, invasive and noninvasive studies have shown that intracranial pressure and related surrogate measures may evolve during the first several days after ROSC, although their magnitude and temporal patterns vary across patients and according to neurologic outcomes [5,7,8]. These observations support serial assessment of intracranial volume compensation but do not establish a direct one-to-one relationship between intracranial pressure and the proportion of CSF volume (pCSFV). On this basis, pCSFV, defined as CSF volume divided by total intracranial volume, has been proposed as a quantitative CT marker of cerebral edema [9,10,11]. However, absolute pCSFV measured at a single time point has shown limited discriminatory performance after cardiac arrest [9], and the reasons for this limitation are likely two-fold: early imaging may precede full evolution of HIBI-related edema, and CSF-space anatomy varies substantially between patients owing to age, sex, cranial size, and pre-existing cerebral atrophy [12,13]. In comatose OHCA survivors, Lee et al. [13] demonstrated that the prognostic performance of ventricular characteristics varied according to age, sex, and CT timing, supporting the concern that single-time-point CSF-space measurements are difficult to interpret without anatomical context.

Serial within-patient assessment may address both limitations by capturing interval changes in pCSFV as a proxy for evolving brain edema and using each patient as their own anatomical reference to partially reduce the influence of inter-individual baseline anatomy. This conceptual basis is well established in ischemic stroke, where reductions in CSF volume between serial scans have been validated as an objective marker of edema progression and poor outcomes [10,11]. In cardiac arrest, although delayed CT and quantitative CT-derived edema metrics have shown improved prognostic performance compared with ultra-early imaging [14,15], the within-patient interval change in pCSFV between early and delayed CT scans obtained within prespecified time windows has not been previously evaluated.

Therefore, we hypothesized that change in pCSFV (ΔpCSFV), defined as delayed pCSFV minus early pCSFV, would reflect progressive CSF-space loss owing to evolving cerebral edema and be associated with 6-month outcomes after adjustment for established clinical, biomarker, and density-based CT predictors. The aims of this study were (i) to test this hypothesis in comatose OHCA survivors treated with targeted temperature management (TTM) and (ii) to assess whether adding ΔpCSFV to a guideline-aligned multimodal baseline model improved discrimination for 6-month outcome.

## 2. Methods

### 2.1. Study Design and Population

This retrospective 72–96-h landmark cohort study was conducted at Chungnam National University Hospital (CNUH), a tertiary referral center in South Korea. We analyzed data from a prospective registry of adult patients with OHCA treated with TTM between May 2019 and April 2025. Seventy-eight patients included in the present study were part of a delayed CT study conducted at the CNUH [14]. The study protocol adhered to the ethical principles of the Declaration of Helsinki and was approved by the Institutional Review Board of Chungnam National University Hospital (CNUH 2025-11-048). The requirement for informed consent was waived because of the retrospective nature of the study and the use of anonymized data.

We included adult patients (≥18 years) who achieved ROSC after non-traumatic OHCA, underwent TTM, and had two non-contrast brain CT scans within predefined time windows: an early CT within 6 h after ROSC and a delayed CT at 72–96 h after ROSC.

Patients were excluded if they had pre-existing major intracranial lesions that could interfere with volumetric analysis, extracorporeal membrane oxygenation (ECMO) use during post-arrest care, incomplete medical records, poor CT image quality precluding quantitative analysis, or failure to undergo both early and delayed CT within the prespecified time windows.

### 2.2. Post-Cardiac Arrest Care and Neurologic Prognostication

All eligible patients were managed in the intensive care unit according to our institutional standard post-resuscitation care protocol [16]. TTM was induced using automated surface or intravascular cooling devices. The target core temperature was set to 33 or 36 °C, maintained for 24 h, followed by controlled rewarming at a rate of 0.25 °C/h. To prevent shivering and minimize cerebral metabolic demand during TTM, continuous infusions of sedatives and neuromuscular blocking agents were administered. At our institution, neurologic prognostication was deferred until after rewarming and no earlier than 72 h after ROSC and was based on a guideline-recommended multimodal approach. During the study period, withdrawal of life-sustaining therapy based on anticipated poor neurologic prognosis was not performed. In patients proceeding to organ donation, life-sustaining treatment was discontinued only after formal determination of brain death. Brain CT findings were not used in isolation for neurologic prognostication or treatment-limitation decisions.

The primary endpoint was neurologic outcome 6 months after cardiac arrest. Neurologic status was assessed using the Cerebral Performance Category (CPC) scale through review of hospital records or direct telephone interviews with patients’ caregivers [17]. Good outcome was defined as CPC 1 or 2, whereas poor outcome was defined as CPC 3, 4, or 5.

### 2.3. Data Collection

We extracted the following data from our institution’s prospective registry: patient characteristics (age, sex, and Charlson Comorbidity Index [CCI]); resuscitation-related variables (witnessed arrest, bystander cardiopulmonary resuscitation [CPR], initial rhythm categorized as shockable versus non-shockable, presumed etiology categorized as cardiac versus non-cardiac, and low-flow time); and variables reflecting post-cardiac arrest illness severity and neurologic injury. The revised Post-Cardiac Arrest Syndrome for Therapeutic Hypothermia (rCAST) score was calculated to quantify post-cardiac arrest syndrome severity. Serum neuron-specific enolase (NSE) level measured 72 h after ROSC was natural-log transformed for multivariable analysis. Pupillary light reflex at 72 h after ROSC was categorized as present or absent. We additionally evaluated whether acute coronary syndrome was diagnosed during the index hospitalization and whether coronary angiography and percutaneous coronary intervention were performed.

### 2.4. Imaging Acquisition and Measurement of Gray-to-White Matter Ratio and pCSFV

At our institution, the standard post-cardiac arrest care protocol recommended brain CT at both prespecified time points—within 6 h and at 72–96 h after ROSC—although imaging at these time points was not mandatory. All patients underwent non-contrast brain CT scans using 64-channel multidetector scanners (Somatom Sensation 16, Somatom Definition AS+, Siemens Healthineers, Forchheim, Germany; or Brilliance 64, Philips Medical Systems, Best, The Netherlands) according to a standard protocol [9]. Images were obtained with a slice thickness of 5 mm. Both early (≤6 h after ROSC) and delayed (72–96 h after ROSC) scans were processed using the same acquisition and reconstruction settings whenever feasible.

#### 2.4.1. Measurement of Gray-to-White Matter Ratio

A board-certified emergency physician, blinded to clinical outcomes, measured Hounsfield units (HU) in the putamen (P), caudate nucleus (CN), posterior limb of the internal capsule (PIC), and corpus callosum (CC) at the basal ganglia level and calculated the gray-to-white matter ratio (GWR) as (P + CN)/(PIC + CC). Circular regions of interest with an area of 9–12 mm^2^ were manually placed over the P, CN, PIC, and CC on each CT scan.

#### 2.4.2. Quantification of pCSFV

**Figure 1:**
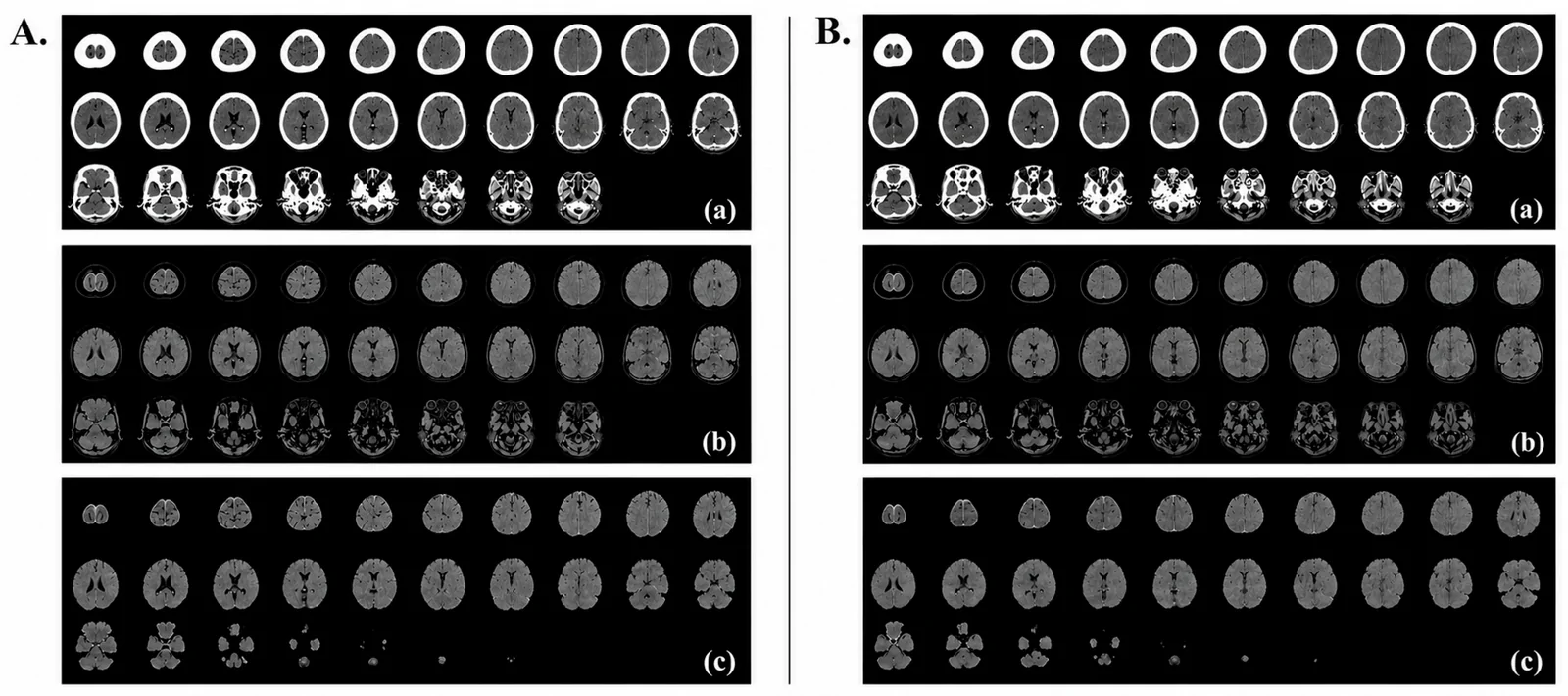
Representative workflow for automated quantitative pCSFV analysis on non-contrast brain CT. (**A**) Early brain CT. (**B**) Delayed brain CT. In each panel, (**a**) original DICOM CT images, (**b**) images after skull removal, and (**c**) segmented cerebrospinal fluid space after removal of extracranial soft tissue are depicted. This representative case was a 78-year-old woman with a poor 6-month outcome. pCSFV decreased from 7.52% on early CT to 4.61% on delayed CT, yielding a ΔpCSFV of −2.91%. Abbreviations: CT, computed tomography; DICOM, Digital Imaging and Communications in Medicine; pCSFV, proportion of cerebrospinal fluid volume; ΔpCSFV, change in pCSFV between delayed and early CT.

Non-contrast brain CT Digital Imaging and Communications in Medicine (DICOM) files were converted to Neuroimaging Informatics Technology Initiative format using MRIcron (http://www.nitrc.org/projects/mricron/, accessed on 7 September 2026). Image processing and HU-based volumetry were performed using the FMRIB Software Library (FSL), Release 5.0 (FMRIB Analysis Group, University of Oxford, Oxford, UK). To restrict analysis to intracranial contents, skull and extracranial soft tissues were removed using FSL, including the Brain Extraction Tool, with a fractional intensity threshold of 0.01, followed by HU-based masking to exclude non-intracranial voxels. Intracranial component voxels were defined using an attenuation range of 0–79 HU, and CSF voxels were segmented using a threshold of 0–15 HU, consistent with prior reports (Figure 1) [18,19]. Voxels outside these predefined ranges were excluded. pCSFV was calculated as CSF volume divided by intracranial component volume. ΔpCSFV was defined as the within-patient difference between delayed and early scans (ΔpCSFV = delayed pCSFV − early pCSFV). All primary pCSFV analyses were performed by a board-certified emergency physician with more than 10 years of experience in quantitative neuroimaging and the custom image-processing pipeline, blinded to clinical data and outcomes. Segmentation outputs were visually inspected, and scans with substantial artifacts or segmentation failures were excluded.

#### 2.4.3. Intraobserver Reliability and Agreement Analysis

To evaluate intraobserver reliability and agreement, 30 patients were randomly selected with stratification by 6-month neurologic outcome: 15 patients with good outcomes and 15 with poor outcomes. The same investigator repeated the complete analysis of de-identified early and delayed CT DICOM data in a separate session without access to the initial measurements. Intraobserver reliability for early pCSFV, delayed pCSFV, and ΔpCSFV was evaluated using two-way mixed-effects, absolute-agreement, single-measurement intraclass correlation coefficients (ICCs) with 95% confidence intervals (CIs). Bland–Altman analyses were additionally performed to estimate the mean difference between repeat and initial measurements and the corresponding 95% limits of agreement. Outcome-stratified analyses were also performed for ΔpCSFV.

### 2.5. Statistical Analysis

No formal a priori sample size calculation was performed. The analytical cohort comprised all consecutive patients in the institutional TTM registry who met the predefined eligibility criteria.

Categorical variables are presented as number (percentage) and were compared using the chi-square or Fisher’s exact test. Continuous variables are presented as mean ± standard deviation or median (interquartile range) and were compared using Student’s *t*-test or the Mann–Whitney U test, as appropriate. Paired early and delayed pCSFV values were compared using the Wilcoxon signed-rank test.

The primary multivariable logistic regression model included five clinically selected predictors: ΔpCSFV, rCAST score, pupillary light reflex at 72 h, natural-log-transformed NSE at 72 h, and delayed CT GWR. The rCAST score represented baseline post-cardiac arrest severity, thereby avoiding separate inclusion of multiple correlated arrest variables. Multicollinearity was assessed using variance inflation factors, and results are reported as adjusted odds ratios (ORs) with 95% CIs.

Missing covariates and their reasons were summarized overall and by neurologic outcome, and missingness was compared between outcome groups using Fisher’s exact test. Fifty imputed datasets were generated using multiple imputation by chained equations. The imputation model included neurologic outcome; all primary-model variables; early and delayed pCSFV; age; sex; witnessed arrest; bystander CPR; and early and delayed CT timing. Neurologic outcome was not imputed. Estimates were pooled using Rubin’s rules, and complete-case analysis was performed as a sensitivity analysis.

Baseline dependence and potential regression-to-the-mean effects were explored using scatterplots and Spearman rank correlations of early pCSFV with delayed pCSFV and ΔpCSFV. Sensitivity models included early pCSFV with either ΔpCSFV or delayed pCSFV and were adjusted using the primary covariate set.

Discrimination was assessed using receiver operating characteristic (ROC) curves and areas under the ROC curve (AUCs) with 95% CIs. The baseline model included rCAST score, pupillary light reflex at 72 h, natural-log-transformed NSE at 72 h, and delayed CT GWR; the extended model additionally included ΔpCSFV. Correlated AUCs were compared using DeLong’s method within each imputed dataset [20], and estimates were pooled across imputations. ROC curves were plotted using participant-level mean predicted probabilities across imputed datasets.

The observed ΔpCSFV cutoff yielding no false-positive results was identified descriptively and considered exploratory because it was data-derived. Sensitivity and specificity were reported with exact binomial 95% CIs. All tests were two-sided, and *p* < 0.05 was considered statistically significant. Analyses were performed using SPSS version 29.0, MedCalc version 21.0, and Python version 3.13.5 (scikit-learn version 1.8.0 and statsmodels version 0.14.6).

## 3. Results

### 3.1. Baseline Characteristics of the Study Cohort

**Figure 2:**
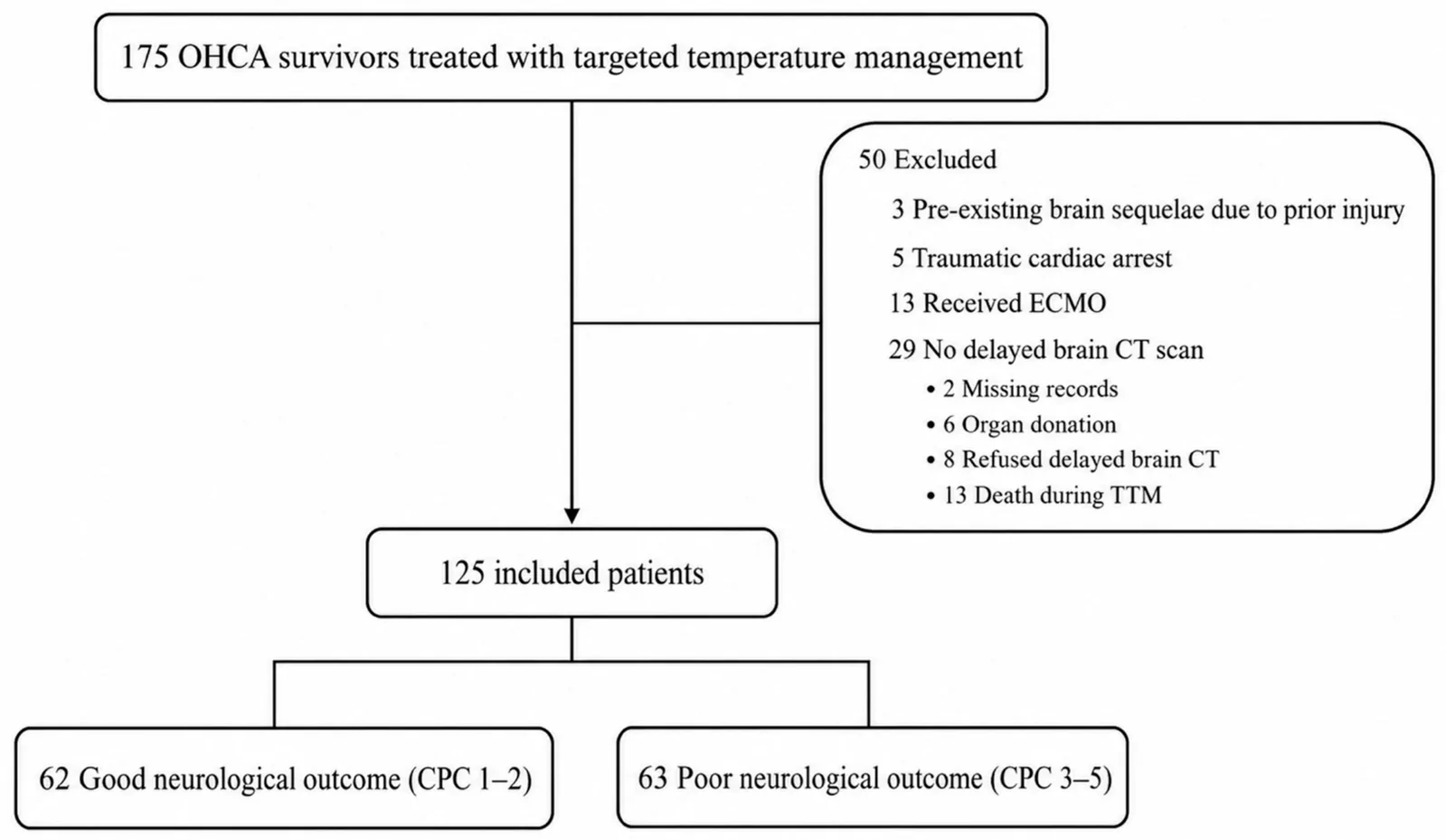
Flow diagram of patient selection. Abbreviations: CPC, Cerebral Performance Category; CT, computed tomography; ECMO, extracorporeal membrane oxygenation; OHCA, out-of-hospital cardiac arrest; TTM, targeted temperature management.

Overall, 175 OHCA survivors underwent TTM during the study period. Of these, 50 were excluded: 3 with pre-existing brain sequelae owing to prior injury, 5 with traumatic cardiac arrest, 13 receiving ECMO, and 29 who did not undergo a second brain CT scan. The final cohort comprised 125 patients who underwent both early and delayed non-contrast brain CT within the predefined time windows. At 6 months, 62 (49.6%) had a good outcome and 63 (50.4%) had a poor outcome (Figure 2).

Baseline demographic and arrest characteristics stratified by 6-month neurologic outcome are summarized in Table 1. Age, sex, CCI, and the interval between early and delayed CT did not differ significantly between outcome groups. Compared with patients with good outcomes, those with poor outcomes were less likely to have favorable arrest characteristics, including witnessed arrest, an initial shockable rhythm, and cardiac etiology. Patients with poor outcomes also had longer low-flow times, higher rCAST scores, and higher serum NSE concentrations. Regarding arrest etiology and coronary evaluation, 64 (51.2%) patients had a presumed cardiac etiology, 30 (24.0%) were diagnosed with acute coronary syndrome, 45 (36.0%) underwent coronary angiography, and 16 (12.8%) underwent percutaneous coronary intervention (Table 1).

Compared with the landmark cohort, patients without delayed CT (n = 29) had a higher rate of poor neurologic outcome (75.9% vs. 50.4%; *p* = 0.020) and a longer median low-flow time (28.0 [18.3–44.5] vs. 20.0 [11.8–31.2] min; *p* = 0.010). Other baseline demographic and arrest characteristics, including rCAST score, did not differ significantly between groups (Table S1).

### 3.2. Association Between pCSFV and Neurologic Outcome

Quantitative CT-derived pCSFV metrics according to 6-month neurologic outcome are summarized in Table 2. Neither early nor delayed pCSFV differed significantly between outcome groups (*p* = 0.280 and *p* = 0.100, respectively). In contrast, ΔpCSFV was significantly lower in the poor-outcome group than in the good-outcome group (*p* < 0.001).

**Figure 3:**
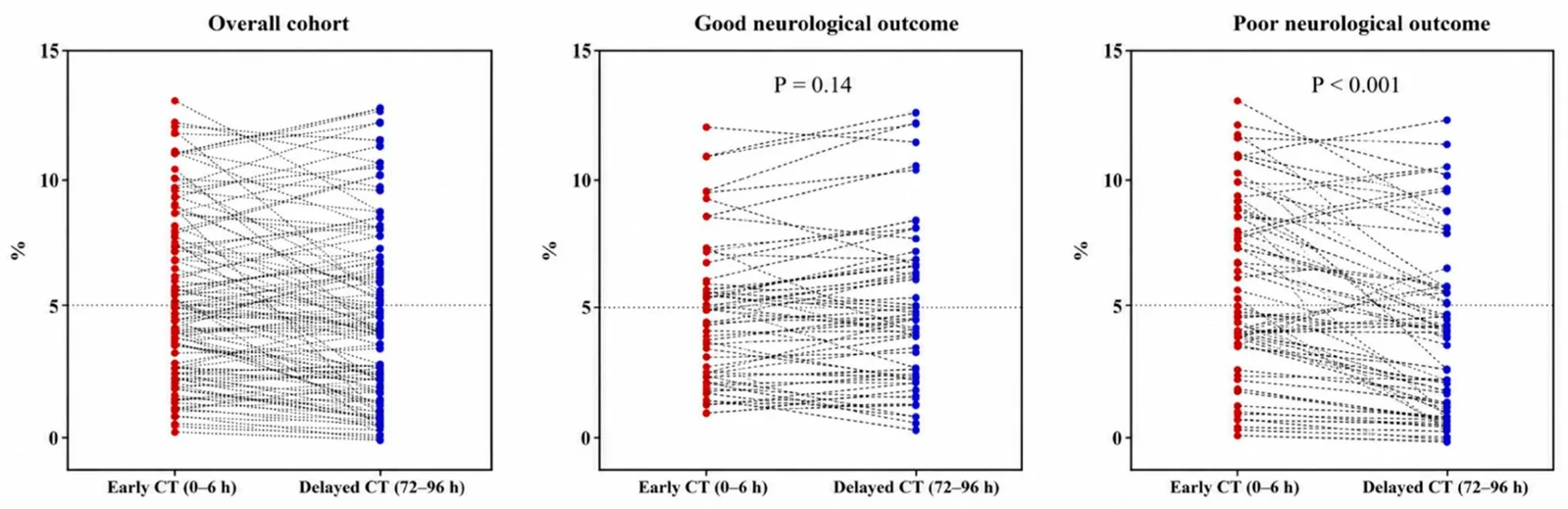
Paired changes in pCSFV between early and delayed brain CT according to neurologic outcome. Red and blue points indicate early and delayed CT measurements, respectively. Paired pCSFV values measured on early CT (0–6 h after ROSC) and delayed CT (72–96 h after ROSC) are shown for the overall cohort and the good- and poor-outcome groups. Each dotted line represents an individual patient. pCSFV did not change significantly in the good-outcome group, whereas it decreased significantly in the poor-outcome group. Abbreviations: CT, computed tomography; pCSFV, proportion of cerebrospinal fluid volume; ROSC, return of spontaneous circulation.

Among patients with good outcomes, the median pCSFV did not differ significantly between early and delayed CT (*p* = 0.140). In contrast, among patients with poor outcomes, median pCSFV was significantly lower on delayed CT than on early CT (*p* < 0.001; Table 2 and Figure 3).

### 3.3. Relationship of Early pCSFV with Delayed pCSFV and ΔpCSFV

Early and delayed pCSFV were strongly correlated overall and within both outcome groups (all ρ ≥ 0.806; *p* < 0.001). Early pCSFV was not significantly correlated with ΔpCSFV overall or in the good-outcome group but showed a modest inverse correlation in the poor-outcome group (ρ = −0.375; *p* = 0.002) (Figure S2 and Table S3).

In the baseline-adjusted sensitivity model using the primary covariate set, the association between ΔpCSFV and poor outcome was attenuated (adjusted OR 0.728, 95% CI 0.462–1.146; *p* = 0.170), whereas early pCSFV remained significant (adjusted OR 1.715, 95% CI 1.173–2.506; *p* = 0.005). The algebraically equivalent model including early and delayed pCSFV yielded similar findings: early pCSFV was associated with poor outcome (adjusted OR 2.355, 95% CI 1.292–4.295; *p* = 0.005), whereas delayed pCSFV was not (adjusted OR 0.728, 95% CI 0.462–1.146; *p* = 0.170) (Table S3).

### 3.4. Intraobserver Reliability and Agreement of pCSFV Measurements

Thirty patients underwent repeat pCSFV analysis, including 15 patients in each neurologic outcome group. Intraobserver reliability was excellent for early pCSFV (ICC 1.000, 95% CI 1.000–1.000), delayed pCSFV (ICC 0.985, 95% CI 0.969–0.993), and ΔpCSFV (ICC 0.976, 95% CI 0.950–0.989). The corresponding mean differences between repeat and initial measurements were −0.004, −0.078, and −0.074 percentage points, respectively. Outcome-stratified ICCs for ΔpCSFV were 1.000 (95% CI 1.000–1.000) in the good-outcome group and 0.939 (95% CI 0.826–0.979) in the poor-outcome group (Table S4).

### 3.5. Missing Data and Multivariable Analysis

Missing values were present for low-flow time in 1 patient, initial shockable rhythm in 1, cardiac etiology in 1, NSE at 72 h in 6, and delayed CT GWR in 6. Across all candidate covariates, 14 patients had at least one missing value; 12 had missingness in at least one primary-model covariate. The proportions of patients with missing primary-model covariates did not differ significantly between the good- and poor-outcome groups (8.1% vs. 11.1%; *p* = 0.763; Table S5).

**Figure 4:**
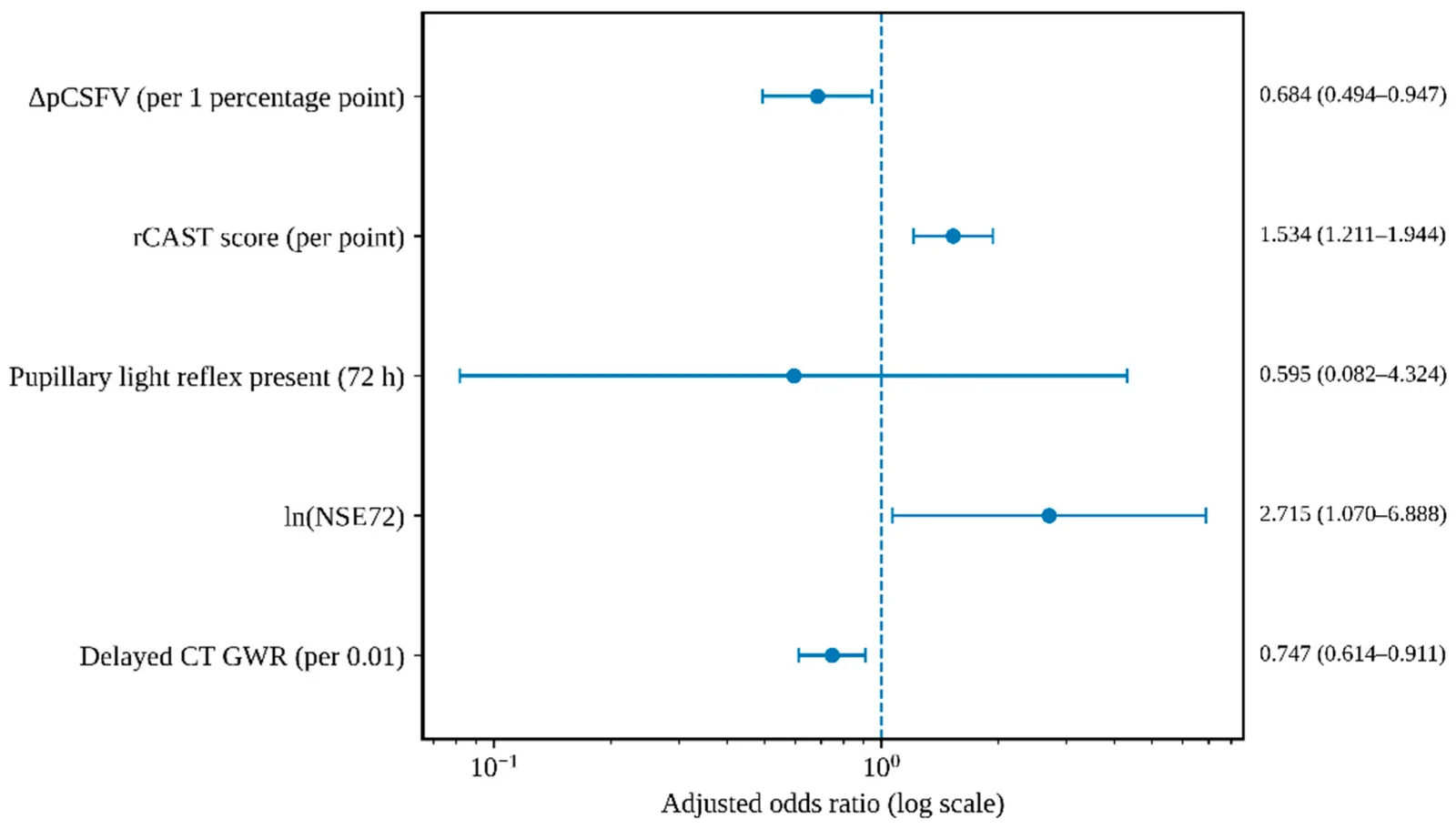
Forest plot of the parsimonious primary multivariable logistic regression model for poor 6-month neurologic outcome. Adjusted odds ratios and 95% CIs were pooled across 50 multiply imputed datasets using Rubin’s rules. The model included ΔpCSFV, rCAST score, pupillary light reflex at 72 h, natural-log-transformed serum NSE at 72 h, and delayed CT GWR. The vertical dashed line indicates an odds ratio of 1, and the x-axis is shown on a logarithmic scale. Abbreviations: CI, confidence interval; CT, computed tomography; GWR, gray-to-white matter ratio; NSE, neuron-specific enolase; OR, odds ratio; pCSFV, proportion of cerebrospinal fluid volume; ΔpCSFV, change in pCSFV between delayed and early CT; rCAST, revised Post-Cardiac Arrest Syndrome for Therapeutic Hypothermia.

The primary multiple-imputation model included all 125 patients, including 63 with poor neurologic outcomes, and contained five predictor parameters. ΔpCSFV was associated with poor outcome (adjusted OR 0.684 per 1 percentage-point increase, 95% CI 0.494–0.947; *p* = 0.022). Higher rCAST score and natural-log-transformed NSE at 72 h and lower delayed CT GWR were also associated with poor outcome (Table S2 and Figure 4). Variance inflation factors were below 2.0 for all predictors.

The complete-case sensitivity analysis included 113 patients, including 56 with poor and 57 with good outcomes. The association between ΔpCSFV and poor outcome was consistent with the primary analysis (adjusted OR 0.648, 95% CI 0.446–0.940; *p* = 0.022) (Table S2).

### 3.6. Discriminatory Performance of pCSFV for Neurologic Outcome

ΔpCSFV alone demonstrated an AUC of 0.722 (95% CI 0.631–0.814) for poor 6-month neurologic outcome. The observed data-derived cutoff of ΔpCSFV ≤ −4.04% yielded a sensitivity of 12.7% (8/63; exact 95% CI 5.6–23.5%) and an observed specificity of 100% (62/62; exact 95% CI 94.2–100.0%). Because the cutoff was derived and evaluated in the same cohort, it was considered exploratory rather than a validated clinical threshold (Figure S1A).

In the multiple-imputation analysis, the baseline model, comprising rCAST score, pupillary light reflex at 72 h, natural-log-transformed NSE at 72 h, and delayed CT GWR, had a pooled AUC of 0.955 (95% CI 0.918–0.991). Addition of ΔpCSFV increased the pooled AUC to 0.962 (95% CI 0.931–0.994), but the difference was not statistically significant (ΔAUC 0.008; *p* = 0.292) (Figure S1B).

## 4. Discussion

The principal finding of this retrospective 72–96-h landmark cohort study was that ΔpCSFV was associated with poor 6-month neurologic outcome in the parsimonious primary multiple-imputation model, whereas neither early nor delayed absolute pCSFV differed significantly between outcome groups. Patients with good outcomes showed little interval change, whereas pCSFV decreased in those with poor outcomes. This association was consistent in the complete-case sensitivity analysis but was attenuated after additional adjustment for early pCSFV, suggesting that baseline dependence may partly contribute to the observed change score.

These findings extend prior work suggesting that single-time-point pCSFV has limited prognostic utility after cardiac arrest [9]. In a retrospective multicenter study, You et al. reported no significant difference in pCSFV between outcome groups on CT acquired at a median of 70 min after ROSC (*p* = 0.900), with overall discriminatory performance remaining poor (AUC 0.521), and attributed this limited performance to the very early imaging window, before HIBI-related cerebral edema had fully evolved [9]. Notably, the early CT in the present study was obtained at a median of 1.0 h after ROSC, which was comparable to the imaging time reported by You et al. [9]. In our cohort, neither early nor delayed absolute pCSFV was significantly associated with outcome, whereas within-patient change was, thereby indicating that absolute pCSFV at a single time point may be influenced not only by the stage of edema evolution but also by inter-individual anatomical variability, including age, sex, cranial size, and pre-existing cerebral atrophy, which may obscure edema-related changes at any given time point [13,21]. Therefore, serial within-patient assessment may provide a more informative marker of evolving cerebral edema, a view supported by Lee et al. [13], who showed that the prognostic performance of ventricular characteristics varied according to age, sex, and CT timing.

The biological plausibility of ΔpCSFV as a marker of post-arrest HIBI is supported by the time course of cerebral edema after cardiac arrest [3]. HIBI evolves over hours to days after ROSC, and progressive brain swelling may reduce the visible CSF compartment before overt intracranial hypertension becomes apparent [3,6]. Unlike GWR or other density-based metrics, which primarily reflect attenuation changes within brain tissue, ΔpCSFV may capture the compensatory reduction in CSF space that accompanies worsening edema [14,15]. Importantly, this study did not assess hydrocephalus or ventricular dilatation. In acute post-arrest HIBI, progressive cerebral edema is expected to compress visible CSF spaces, including the sulci and ventricles, rather than cause hydrocephalus. Ventricular enlargement on a single CT may instead reflect baseline anatomy, age-related atrophy, sex, or scan timing. NSE and ΔpCSFV represent distinct but complementary domains—neuronal injury and structural CSF-space change—and should not be considered interchangeable or causally linked.

From a clinical perspective, the contribution of ΔpCSFV should be interpreted in the context of multimodal neuroprognostication. The baseline model already showed high discriminatory performance (AUC 0.955), and adding ΔpCSFV did not significantly improve discrimination (AUC 0.962; ΔAUC 0.008; *p* = 0.292). This finding should be interpreted cautiously because AUC-based comparisons may be relatively insensitive to the contribution of an additional marker when baseline discrimination is already high [22].

ΔpCSFV should be interpreted as a complementary serial measure rather than a baseline-independent marker. It was associated with poor outcome in the parsimonious primary model, but additional adjustment for early pCSFV attenuated the association. Thus, within-patient change may reflect temporal evolution of cerebral edema while partially reducing inter-individual anatomical variability; however, mathematical coupling, baseline dependence, and possible regression-to-the-mean effects may also contribute. In this respect, ΔpCSFV may complement density-based markers such as GWR, which primarily reflect attenuation changes within brain tissue rather than compensatory CSF displacement.

Overall, these findings support further evaluation of ΔpCSFV as an adjunctive serial imaging marker of evolving HIBI within multimodal neuroprognostication. These findings apply only to patients who survived and underwent delayed CT at 72–96 h. The observed data-derived cutoff (ΔpCSFV ≤ −4.04%) is exploratory and requires external validation before any clinical application.

This study has several limitations. First, this was a single-center retrospective 72–96-h landmark study with a modest sample size. Patients without delayed CT had longer low-flow times and more frequent poor neurologic outcomes, indicating potential survivor selection bias. Accordingly, the findings are conditional on survival to and completion of delayed imaging at 72–96 h and should not be extrapolated to patients who died before the landmark time point. Second, although the parsimonious model reduced the number of predictor parameters and multiple imputation allowed all 125 patients to be included in the primary analysis, residual overfitting cannot be excluded. Moreover, multiple imputation relies on a missing-at-random assumption conditional on the observed data, and bias related to unmeasured determinants of missingness may remain. Third, although intraobserver reliability was excellent for early, delayed, and interval-change pCSFV measurements, interobserver reliability was not evaluated. CT-based pCSFV quantification may also be affected by scanner parameters and segmentation methodology; therefore, reproducibility across analysts and institutions remains to be established. Fourth, the target temperature was set at either 33 or 36 °C at the treating team’s discretion, and potential differential effects of TTM temperature on cerebral edema dynamics could not be excluded. Fifth, the ΔpCSFV cutoff yielding no false-positive results was derived and evaluated in the same cohort and may therefore be unstable and optimistically estimated. It should not be interpreted as a validated clinical threshold without external validation. Finally, although imaging analyses were performed retrospectively and blinded to outcome, treating clinicians had access to CT findings; therefore, a self-fulfilling prophecy effect cannot be entirely excluded, even though prognostication at our institution followed a guideline-based multimodal protocol applied no earlier than 72 h after ROSC [23]. Collectively, these findings should be interpreted as hypothesis-generating pending external validation.

## 5. Conclusions

Among comatose OHCA survivors who underwent delayed CT at the 72–96-h landmark, ΔpCSFV was associated with poor 6-month neurologic outcome in the parsimonious primary multiple-imputation model, with consistent findings in the complete-case sensitivity analysis. However, attenuation after adjustment for early pCSFV indicates partial dependence on the baseline measurement. ΔpCSFV may therefore provide complementary information on serial CSF-space change within multimodal neuroprognostication but should not be interpreted as a baseline-independent marker. These findings apply only to patients who survived and underwent delayed CT at 72–96 h and should not be extrapolated to patients who died before delayed imaging. The observed data-derived cutoff is exploratory and requires external multicenter validation before any clinical application.

## Figures and Tables

**Table 1:** Clinical and arrest characteristics according to 6-month neurologic outcome.

Characteristics	Cohort (n = 125)	Good Neurologic Outcome (n = 62)	Poor Neurologic Outcome (n = 63)	*p*-Value ^a^
Age, years	61.0 (49.0–70.0)	61.0 (48.8–66.0)	64.0 (49.5–73.0)	0.170
Male sex	94 (75.2)	51 (82.3)	43 (68.3)	0.110
Charlson Comorbidity Index score	3.0 (1.0–4.0)	2.0 (1.0–4.0)	3.0 (1.0–5.0)	0.200
Arrest characteristics				
Witnessed arrest	79 (63.2)	49 (79.0)	30 (47.6)	<0.001
Bystander CPR	88 (70.4)	47 (75.8)	41 (65.1)	0.264
Shockable rhythm	54 (43.2)	43 (69.4)	11 (17.5)	<0.001
Cardiac etiology	64 (51.2)	46 (74.2)	18 (28.6)	<0.001
Low-flow time, min	20.0 (11.8–31.2)	15.0 (9.0–22.0)	26.0 (19.5–36.5)	<0.001
rCAST score	11.0 (7.5–14.5)	7.5 (5.0–10.9)	14.0 (12.0–16.2)	<0.001
Coronary evaluation and intervention				
Acute coronary syndrome	30 (24.0)	23 (37.1)	7 (11.1)	0.001
Coronary angiography	45 (36.0)	40 (64.5)	5 (7.9)	<0.001
Percutaneous coronary intervention	16 (12.8)	14 (22.6)	2 (3.2)	0.001
Pupillary light reflex present at 72 h after ROSC	96 (76.8)	58 (93.5)	38 (60.3)	<0.001
Serum NSE, ng/mL				
72 h after ROSC	26.9 (16.4–82.5)	17.1 (13.4–26.8)	82.0 (32.7–182.5)	<0.001
GWR				
Early CT	1.12 (1.10–1.13)	1.13 (1.11–1.15)	1.11 (1.08–1.12)	<0.001
Delayed CT	1.11 (1.04–1.15)	1.14 (1.12–1.16)	1.04 (1.01–1.09)	<0.001
Time from ROSC to scan, h				
Early CT	1.0 (0.6–1.9)	0.9 (0.6–1.6)	1.1 (0.6–2.2)	0.570
Delayed CT	77.8 (75.9–79.4)	77.7 (75.5–79.2)	78.1 (76.3–79.7)	0.260

Continuous variables are presented as median (interquartile range) and categorical variables as number (%). ^a^ *p*-values were calculated using the chi-square test for categorical variables or Fisher’s exact test and the Mann–Whitney U test for continuous variables. Abbreviations: CPR, cardiopulmonary resuscitation; CT, computed tomography; GWR, gray-to-white matter ratio; NSE, neuron-specific enolase; rCAST, revised Post-Cardiac Arrest Syndrome for Therapeutic Hypothermia; ROSC, return of spontaneous circulation.

**Table 2:** Comparison of early, delayed, and interval change in pCSFV according to 6-month neurologic outcome.

**Variable**	**Good Neurologic Outcome** **(N = 62)**	**Poor Neurologic Outcome (N = 63)**	***p*** **-Value ^a^**
Early pCSFV (%)	4.55 (2.66–5.97)	4.88 (3.00–8.43)	0.280
Delayed pCSFV (%)	4.55 (2.64–6.70)	4.09 (1.44–6.14)	0.100
ΔpCSFV (%)	0.30 (−0.55 to 0.92)	−0.83 (−2.58 to −0.16)	<0.001
**Outcome Group**	**Early pCSFV (%)**	**Delayed pCSFV (%)**	***p*** **-Value ^b^**
Good neurologic outcome	4.55 (2.66–5.97)	4.55 (2.64–6.70)	0.140
Poor neurologic outcome	4.88 (3.00–8.43)	4.09 (1.44–6.14)	<0.001

Continuous variables are presented as median (interquartile range). ^a^ *p*-values were calculated using the Mann–Whitney U test. ^b^ *p*-values were calculated using the Wilcoxon signed-rank test. Abbreviation: pCSFV, proportion of cerebrospinal fluid volume; ΔpCSFV, change in pCSFV between delayed and early CT.

## Data Availability

The data are available from the corresponding author upon reasonable request. The data are not publicly available because they contain sensitive clinical information.

## Supplementary Materials

The following supporting information can be downloaded at https://www.mdpi.com/article/10.3390/jcm15186978/s1, Figure S1, Receiver operating characteristic curves for ΔpCSFV and the multivariable models; Figure S2, Scatterplots showing the relationships of early pCSFV with delayed pCSFV and with ΔpCSFV; Table S1, Comparison of baseline demographic and arrest characteristics and 6-month neurologic outcome between the 72–96-h landmark cohort and patients without delayed CT; Table S2, Parsimonious multivariable model for poor 6-month neurologic outcome; Table S3, Correlation analyses and sensitivity multivariable models examining baseline dependence of ΔpCSFV; Table S4, Intraobserver reliability and agreement of early, delayed, and interval-change pCSFV measurements; Table S5, Extent, distribution, and reasons for missing covariate data.

## Abbreviations

The following abbreviations are used in this manuscript:

AUC	Area under the curve
CC	Corpus callosum
CCI	Charlson Comorbidity Index
CI	Confidence interval
CN	Caudate nucleus
CNUH	Chungnam National University Hospital
CPC	Cerebral Performance Category
CPR	Cardiopulmonary resuscitation
CSF	Cerebrospinal fluid
CT	Computed tomography
DICOM	Digital Imaging and Communications in Medicine
ECMO	Extracorporeal membrane oxygenation
FSL	FMRIB Software Library
GWR	Gray-to-white matter ratio
HIBI	Hypoxic–ischemic brain injury
HU	Hounsfield unit
ICC	Intraclass correlation coefficient
NSE	Neuron-specific enolase
OHCA	Out-of-hospital cardiac arrest
OR	Odds ratio
P	Putamen
pCSFV	Proportion of cerebrospinal fluid volume
ΔpCSFV	Change in pCSFV between delayed and early CT
PIC	Posterior limb of the internal capsule
rCAST	Revised Post-Cardiac Arrest Syndrome for Therapeutic Hypothermia
ROC	Receiver operating characteristic
ROSC	Return of spontaneous circulation
TTM	Targeted temperature management

## References

[1] Nolan J.P., Sandroni C., Cariou A., Cronberg T., D’Arrigo S., Haywood K., Hoedemaekers A., Lilja G., Nikolaou N., Olasveengen T.M. (2025). European Resuscitation Council and European Society of Intensive Care Medicine guidelines 2025 Post-resuscitation care. Resuscitation.

[2] Sandroni C., D’Arrigo S., Nolan J.P. (2018). Prognostication after cardiac arrest. Crit. Care.

[3] Sandroni C., Cronberg T., Sekhon M. (2021). Brain injury after cardiac arrest: Pathophysiology, treatment, and prognosis. Intensive Care Med..

[4] Jeon S.Y., Min J.H., Park J.S., Kang C., You Y., Jeong W., Ryu H.S., Lim J.A., Lee B.K. (2025). Time-resolved ADC analysis differentiates stable vs. progressive brain injury in post-cardiac arrest patients. Resuscitation.

[5] Song H., Kang C., Park J., You Y., In Y., Min J., Jeong W., Cho Y., Ahn H., Kim D. (2021). Intracranial pressure patterns and neurological outcomes in out-of-hospital cardiac arrest survivors after targeted temperature management: A retrospective observational study. J. Clin. Med..

[6] Mokri B. (2001). The Monro-Kellie hypothesis: Applications in CSF volume depletion. Neurology.

[7] Sekhon M.S., Griesdale D.E., Ainslie P.N., Gooderham P., Foster D., Czosnyka M., Robba C., Cardim D. (2019). Intracranial pressure and compliance in hypoxic ischemic brain injury patients after cardiac arrest. Resuscitation.

[8] Park J.S., Cho Y., You Y., Min J.H., Jeong W., Ahn H.J., Kang C., Yoo I., Ryu S., Lee J. (2019). Optimal timing to measure optic nerve sheath diameter as a prognostic predictor in post-cardiac arrest patients treated with targeted temperature management. Resuscitation.

[9] You Y.H., Park J.S., Yoo I.S., Min J.H., Jeong W.J., Cho Y.C., Ryu S., Lee J.W., Kim S.W., Cho S.U. (2019). Usefulness of a quantitative analysis of the cerebrospinal fluid volume proportion in brain computed tomography for predicting neurological prognosis in cardiac arrest survivors who undergo target temperature management. J. Crit. Care.

[10] Dhar R., Chen Y., Hamzehloo A., Kumar A., Heitsch L., He J., Chen L., Slowik A., Strbian D., Lee J.M. (2020). Reduction in cerebrospinal fluid volume as an early quantitative biomarker of cerebral edema after ischemic stroke. Stroke.

[11] Dhar R., Yuan K., Kulik T., Chen Y., Heitsch L., An H., Ford A., Lee J.M. (2016). CSF volumetric analysis for quantification of cerebral edema after hemispheric infarction. Neurocrit. Care.

[12] Hong J.Y., Lee D.H., Oh J.H., Lee S.H., Choi Y.H., Kim S.H., Min J.H., Kim S.J., Park Y.S., Korean Hypothermia Network Investigators (2019). Grey-white matter ratio measured using early unenhanced brain computed tomography shows no correlation with neurologic outcomes in patients undergoing targeted temperature management after cardiac arrest. Resuscitation.

[13] Lee B.K., Callaway C.W., Coppler P.J., Rittenberger J.C., Pittsburgh Post-Cardiac Arrest Service (2020). The prognostic performance of brain ventricular characteristic differ according to sex, age, and time after cardiac arrest in comatose out-of-hospital cardiac arrest survivors. Resuscitation.

[14] In Y.N., Lee I.H., Park J.S., Kim D.M., You Y., Min J.H., Jeong W., Ahn H.J., Kang C., Lee B.K. (2022). Delayed head CT in out-of-hospital cardiac arrest survivors: Does this improve predictive performance of neurological outcome?. Resuscitation.

[15] In Y.N., Kim H.I., Park J.S., Kang C., You Y., Min J.H., Lee D., Lee I.H., Jeong H.S., Lee B.K. (2024). Association between quantitative analysis of cerebral edema using CT imaging and neurological outcomes in cardiac arrest survivors. Am. J. Emerg. Med..

[16] Jeon G.R., Ahn H.J., Park J.S., Yoo I., You Y., Cho Y.C., Jeong W., Kang C., Lee B.K. (2021). The association between neurological prognosis and the degree of blood–brain barrier disruption in cardiac arrest survivors who underwent target temperature management. Neurocrit. Care.

[17] You Y., Park J.S., Min J., Yoo I., Ahn H.J., Cho Y., Ryu S., Lee J., Kim S., Cho S. (2019). The usefulness of neuron-specific enolase in cerebrospinal fluid to predict neurological prognosis in cardiac arrest survivors who underwent target temperature management: A prospective observational study. Resuscitation.

[18] Hagen T., Ahlhelm F., Reiche W. (2007). Apparent diffusion coefficient in vasogenic edema and reactive astrogliosis. Neuroradiology.

[19] Pappu S., Lerma J., Khraishi T. (2016). Brain CT to assess intracranial pressure in patients with traumatic brain injury. J. Neuroimaging.

[20] DeLong E.R., Delong D.M., Clarke-Pearson D.L. (1988). Comparing the areas under two or more correlated receiver operating characteristic curves: A nonparametric approach. Biometrics.

[21] Kaye J.A., DeCarli C., Luxenberg J.S., Rapoport S.I. (1992). The significance of age-related enlargement of the cerebral ventricles in healthy men and women measured by quantitative computed X-ray tomography. J. Am. Geriatr. Soc..

[22] Pencina M.J., D’Agostino R.B., D’Agostino R.B., Vasan R.S. (2008). Evaluating the added predictive ability of a new marker: From area under the ROC curve to reclassification and beyond. Stat. Med..

[23] Elmer J., Torres C., Aufderheide T.P., Austin M.A., Callaway C.W., Golan E., Herren H., Jasti J., Kudenchuk P.J., Scales D.C. (2016). Association of early withdrawal of life-sustaining therapy for perceived neurological prognosis with mortality after cardiac arrest. Resuscitation.

