# Supplementary material for: Serial Changes in CSF Volume Proportion on Brain CT and 6-Month Neurologic Outcomes After Cardiac Arrest: A 72–96-h Landmark Cohort Study
Source: J Clin Med. 2026 Sep 9;15(18):6978. doi: 10.3390/jcm15186978 (PMC13607377; doi:10.3390/jcm15186978)
Supplement: Supplementary file 1 [file jcm-15-06978-s001.zip › jcm-4425589-supplementary.pdf]

Supplemental Online Content

Serial Change in CSF Volume Proportion on Brain CT and 6-Month Neurologic Outcome After Cardiac Arrest: A 72–96 h Landmark Cohort Study

Table S1. Comparison of baseline demographic and arrest characteristics and 6-month neurologic outcome between the 72–96 h landmark cohort and patients without delayed CT

Table S2. Parsimonious multivariable model for poor 6-month neurologic outcome

Table S3. Correlation analyses and sensitivity multivariable models examining baseline dependence of ΔpCSFV

Table S4. Intraobserver reliability and agreement of early, delayed, and interval-change pCSFV measurements

Table S5. Extent, distribution, and reasons for missing covariate data

Figure S1. Receiver operating characteristic curves for ΔpCSFV and the multivariable models

Figure S2. Scatterplots showing the relationships of early pCSFV with delayed pCSFV and with ΔpCSFV

Table S1. Comparison of baseline demographic and arrest characteristics and 6-month neurologic outcome between the 72–96 h landmark cohort and patients without delayed CT

Characteristics	Landmark cohort (n = 125)	No delayed CT (n = 29)	P valuea
Age, years	61.0 (49.0–70.0)	60.0 (44.8–71.8)	0.930
Male sex	94 (75.2)	20 (69.0)	0.640
Charlson Comorbidity Index score	3.0 (1.0–4.0)	2.5 (1.0–5.5)	0.970
Arrest characteristics			
Witnessed arrest	79 (63.2)	14 (48.3)	0.140
Bystander CPR	88 (70.4)	18 (62.1)	0.510
Shockable rhythm	54 (43.2)	12 (41.4)	0.840
Cardiac etiology	64 (51.2)	16 (55.2)	0.690
Low-flow time, min	20.0 (11.8–31.2)	28.0 (18.3–44.5)	0.010
rCAST score	11.0 (7.5–14.5)	12.5 (7.6–16.1)	0.650
Neurologic outcome			
Poor neurologic outcome	63 (50.4)	22 (75.9)	0.013

Continuous variables are presented as median (interquartile range) and categorical variables as number (%).

a P values were calculated using the chi-square test or Fisher's exact test for categorical variables and the Mann–Whitney U test for continuous variables.

Abbreviations: CPR, cardiopulmonary resuscitation; rCAST, revised Post-Cardiac Arrest Syndrome for Therapeutic Hypothermia.

Table S2. Parsimonious multivariable model for poor 6-month neurologic outcome

Variable	Primary multiple-imputation model, adjusted OR (95% CI)	P value	Complete-case sensitivity model, adjusted OR (95% CI)	P value
ΔpCSFV, per 1 percentage-point increase	0.684 (0.494–0.947)	0.022	0.648 (0.446–0.940)	0.022
rCAST score, per 1-point increase	1.534 (1.211–1.944)	< 0.001	1.516 (1.187–1.938)	< 0.001
Pupillary light reflex present at 72 h	0.595 (0.082–4.324)	0.608	1.038 (0.118–9.127)	0.973
ln(NSE72)	2.715 (1.070–6.888)	0.036	3.219 (1.157–8.951)	0.025
Delayed CT GWR, per 0.01 increase	0.747 (0.614–0.911)	0.004	0.730 (0.594–0.897)	0.003

The primary model included five clinically selected predictor parameters: ΔpCSFV, rCAST score, pupillary light reflex at 72 h, natural-log-transformed serum NSE at 72 h, and delayed CT GWR.

Missing covariates were handled using multiple imputation by chained equations with 50 imputed datasets; estimates were pooled using Rubin's rules. The primary analysis included all 125 patients, including 63 with poor neurologic outcomes (12.6 poor-outcome events per predictor parameter).

The complete-case sensitivity analysis included 113 patients, including 56 with poor and 57 with good neurologic outcomes (11.2 poor-outcome events per predictor parameter).

The imputation model included neurologic outcome; all primary-model variables; early and delayed pCSFV; age; sex; witnessed arrest; bystander CPR; and early and delayed CT timing.

Adjusted ORs for pCSFV are reported per 1 percentage-point increase. All P values are two-sided.

Abbreviations: CI, confidence interval; CT, computed tomography; GWR, gray-to-white matter ratio; NSE, neuron-specific enolase; OR, odds ratio; pCSFV, proportion of cerebrospinal fluid volume; rCAST, revised Post-Cardiac Arrest Syndrome for Therapeutic Hypothermia.

Table S3A. Spearman correlations between early and delayed pCSFV and between early pCSFV and ΔpCSFV

Group	n	Early vs. delayed pCSFV, ρ	P value	Early pCSFV vs. ΔpCSFV, ρ	P value
Overall cohort	125	0.817	< 0.001	−0.154	0.087
Good neurologic outcome	62	0.894	< 0.001	0.171	0.185
Poor neurologic outcome	63	0.806	< 0.001	−0.375	0.002

Correlations were calculated using Spearman rank correlation in the overall cohort and after stratification by 6-month neurologic outcome. ΔpCSFV was defined as delayed pCSFV minus early pCSFV. All P values are two-sided.

Abbreviations: CT, computed tomography; pCSFV, proportion of cerebrospinal fluid volume; ΔpCSFV, change in pCSFV between delayed and early CT.

Table S3B. Baseline-dependence sensitivity models using the primary covariate set

Model	Variable	Adjusted OR	95% CI	P value
Model 1: Early pCSFV + ΔpCSFV	Early pCSFV, per 1 percentage-point increase	1.715	1.173–2.506	0.005
	ΔpCSFV, per 1 percentage-point increase	0.728	0.462–1.146	0.170
Model 2: Early pCSFV + delayed pCSFV	Early pCSFV, per 1 percentage-point increase	2.355	1.292–4.295	0.005
	Delayed pCSFV, per 1 percentage-point increase	0.728	0.462–1.146	0.170

Both models used multiple imputation by chained equations with 50 imputed datasets and included all 125 patients.

Both models were adjusted for rCAST score, pupillary light reflex at 72 h, natural-log-transformed serum NSE at 72 h, and delayed CT GWR. Only pCSFV coefficients are displayed.

Because ΔpCSFV = delayed pCSFV − early pCSFV, Models 1 and 2 are algebraically equivalent parameterizations containing the same information and have identical model fit.

Adjusted ORs for pCSFV variables are reported per 1 percentage-point increase. All P values are two-sided.

Abbreviations: CI, confidence interval; CT, computed tomography; GWR, gray-to-white matter ratio; NSE, neuron-specific enolase; OR, odds ratio; pCSFV, proportion of cerebrospinal fluid volume; rCAST, revised Post-Cardiac Arrest Syndrome for Therapeutic Hypothermia.

Table S4. Intraobserver reliability and agreement of early, delayed, and interval-change pCSFV measurements

Measurement	n	ICC (95% CI)	Mean difference (repeat - initial), percentage points	95% limits of agreement, percentage points
**Panel A. Overall intraobserver reliability and agreement (n = 30)**				
Early pCSFV	30	1.000 (1.000–1.000)	−0.004	−0.090 to 0.082
Delayed pCSFV	30	0.985 (0.969–0.993)	−0.078	−0.970 to 0.815
ΔpCSFV	30	0.976 (0.950–0.989)	−0.074	−0.978 to 0.830
**Panel B. Outcome-stratified intraobserver reliability and agreement for ΔpCSFV**				
Good neurologic outcome	15	1.000 (1.000–1.000)	0.003	−0.026 to 0.031
Poor neurologic outcome	15	0.939 (0.826–0.979)	−0.150	−1.432 to 1.132

Notes: Thirty patients were randomly selected with stratification by 6-month neurologic outcome, including 15 patients with good outcomes and 15 with poor outcomes. The same experienced investigator repeated the complete analysis of de-identified early and delayed CT DICOM data in a separate session without access to the initial measurements. Intraclass correlation coefficients and 95% confidence intervals were calculated using a two-way mixed-effects, absolute-agreement, single-measurement model. Bland–Altman mean differences were calculated as repeat minus initial measurements, and the 95% limits of agreement were defined as the mean difference ± 1.96 standard deviations of the paired differences. Mean differences and limits of agreement are expressed in percentage points.

Abbreviations: CI, confidence interval; CT, computed tomography; DICOM, Digital Imaging and Communications in Medicine; ICC, intraclass correlation coefficient; pCSFV, proportion of cerebrospinal fluid volume; ΔpCSFV, change in pCSFV between delayed and early CT.

Table S5. Extent, distribution, and reasons for missing data

Variable	Overall, n/N (%)	Good outcome, n/N (%)	Poor outcome, n/N (%)	P value	Reason for missingness
Low-flow time	1/125 (0.8)	1/62 (1.6)	0/63 (0.0)	0.496	Exact CPR-to-ROSC time could not be confirmed.
Initial shockable rhythm	1/125 (0.8)	1/62 (1.6)	0/63 (0.0)	0.496	Initial rhythm was not documented.
Cardiac etiology	1/125 (0.8)	1/62 (1.6)	0/63 (0.0)	0.496	Reason not documented in the current dataset.
NSE at 72 h	6/125 (4.8)	2/62 (3.2)	4/63 (6.3)	0.680	Blood sampling was not performed.
Delayed CT GWR	6/125 (4.8)	3/62 (4.8)	3/63 (4.8)	1.000	ROI measurement was not feasible.
Any candidate covariate	14/125 (11.2)	7/62 (11.3)	7/63 (11.1)	1.000	—
Any primary-model covariate	12/125 (9.6)	5/62 (8.1)	7/63 (11.1)	0.763	—

P values were calculated using Fisher's exact test.

Patients with more than one missing variable were counted once in the rows for any candidate or primary-model covariate.

The primary-model covariates with missing data were NSE at 72 h and delayed CT GWR; low-flow time and initial shockable rhythm were included only in the original candidate set.

Abbreviations: CPR, cardiopulmonary resuscitation; CT, computed tomography; GWR, gray-to-white matter ratio; NSE, neuron-specific enolase; ROI, region of interest; ROSC, return of spontaneous circulation.

Figure S1. Receiver operating characteristic curves for ΔpCSFV and the multivariable models

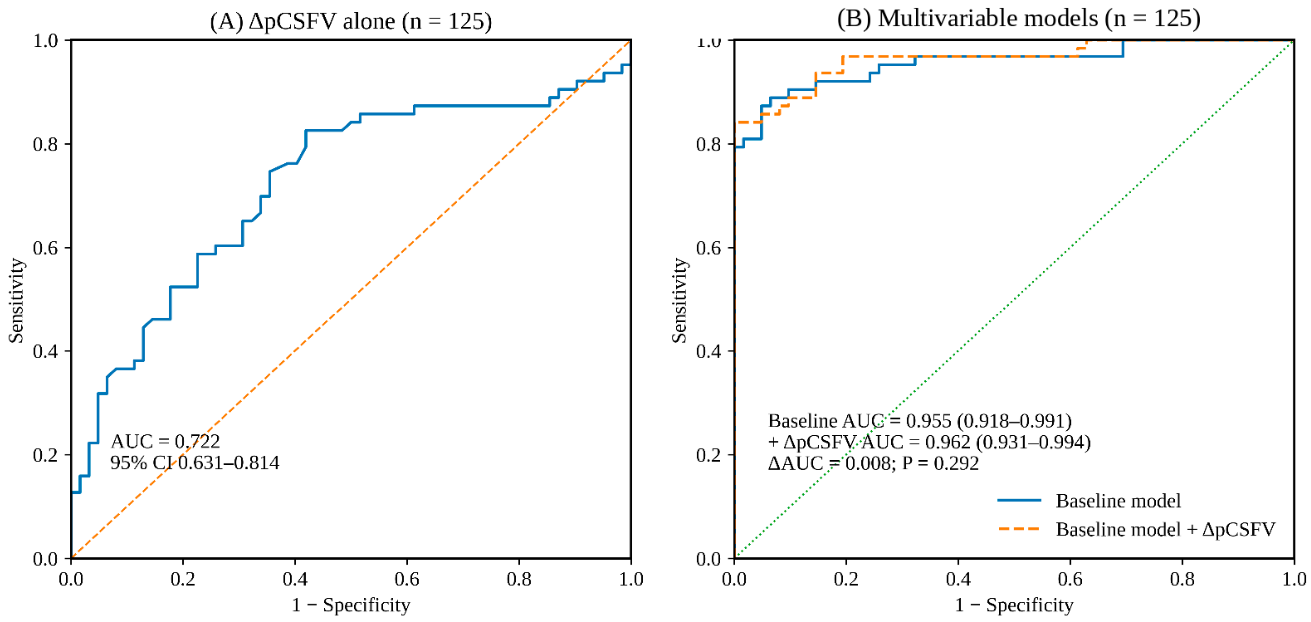

(A) ROC curve for ΔpCSFV alone. (B) ROC curves for the baseline model and the baseline model plus ΔpCSFV. The baseline model included rCAST score, pupillary light reflex at 72 h, natural-log-transformed serum NSE at 72 h, and delayed CT GWR; the extended model additionally included ΔpCSFV. Curves were generated from participant-level mean predicted probabilities across 50 imputed datasets, and AUC estimates and comparisons were pooled across imputations.

Abbreviations: AUC, area under the curve; CI, confidence interval; CT, computed tomography; GWR, gray-to-white matter ratio; NSE, neuron-specific enolase; pCSFV, proportion of cerebrospinal fluid volume; ROC, receiver operating characteristic.

Figure S2. Scatterplots showing the relationships of early pCSFV with delayed pCSFV and with ΔpCSFV

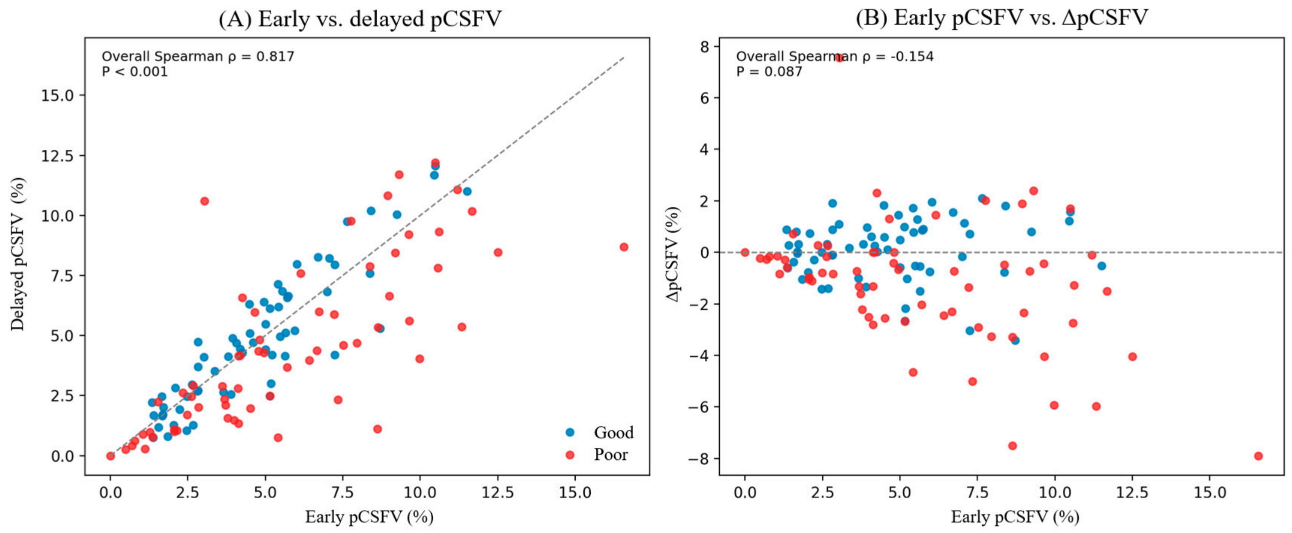

(A) Relationship between early and delayed pCSFV. Early and delayed pCSFV showed a strong positive correlation overall and within both neurologic outcome groups. (B) Relationship between early pCSFV and ΔpCSFV. The correlation between early pCSFV and ΔpCSFV was weak in the overall cohort and in the good-outcome group, whereas a modest inverse correlation was observed in the poor-outcome group. Blue and red points represent good and poor neurologic outcomes, respectively.

Abbreviations: CT, computed tomography; pCSFV, proportion of cerebrospinal fluid volume.
